# Microwave ablation of thyroid nodules and metastatic thyroid cervical lymph nodes: the first case series from Ecuador

**DOI:** 10.1093/jscr/rjaf197

**Published:** 2025-04-07

**Authors:** Richard Godoy, Paola Solis-Pazmino, Fernando Walder, Eddy P Lincango, Carlos Lehn

**Affiliations:** Head and Neck Surgery, Hospital Metropolitano, Quito 170508, Ecuador; Medical Director, Clínica de Tiroides y Cuello, Quito, Ecuador; Surgery Group of Los Angeles, Los Angeles, CA 90048, United States; Surgery Department, Santa Casa de Porto Alegre, Porto Alegre, Rio Grande do Sul 90020-090, Brazil; CaTaLiNA-Cancer de Tiroides en Latino America, Quito, Pichincha, Ecuador; Head and Neck Surgery, Universidade Federal de São Paulo, Escola Paulista de Medicina, São Paulo, SP, Brazil; CaTaLiNA-Cancer de Tiroides en Latino America, Quito, Pichincha, Ecuador; Head and Neck Surgery, Hospital do Servidor Público Estadual de São Paulo, São Paulo, Brazil

**Keywords:** microwave ablation, thyroid nodules, thyroid cancer

## Abstract

Microwave ablation (MWA) has emerged as a minimally invasive technique, transforming the therapeutic landscape for benign thyroid nodules (BTN), papillary thyroid microcarcinoma (PTMC), and cervical lymph node metastasis (CLNM). This study aims to present the first experience in Ecuador using MWA. We included adults undergoing MWA for BTN, PTMC, and CLNM between September 2022 and April 2023. Descriptive statistics and the Wilcoxon signed-rank test were used to compare some pre- and post-intervention outcomes. Fourteen patients (11 BTN, one PMTC, and two CLNM) were included. Most were female (86%; mean age 48.92 years) and euthyroid (73%). Most nodules were solid (92%). The volume reduction (VR) of BTN was 98% at 12-months. The PTMC nodule disappeared and the CLNM mean volume reduced to 0.03 at 12 months. These findings highlight MWA as a potential treatment for various thyroid pathologies, warranting further exploration and validation in larger cohorts.

## Introduction

The prevalence of thyroid nodules (TN) is on the rise globally, primarily comprising benign thyroid nodules (BTN) with a smaller subset diagnosed as papillary thyroid microcarcinoma (PTMC) [1]. Approximately one-third of BTN cases necessitate therapeutic intervention, often driven by symptomatic or cosmetic concerns arising from nodule enlargement.

Historically, surgical treatment has been the cornerstone of TN management [2]. However, drawbacks such as scarring and the need for thyroid hormone replacement therapy have prompted patients to seek alternative minimally invasive techniques (MIT) [3] including transoral endoscopic thyroidectomy via vestibular approach, transoral robotic surgery or ablation techniques like ethanol, laser, high intensity focused ultrasound, radiofrequency, and microwave ablation (MWA). MWA can be applied for both benign (cystic and solid) TN and PTMC [4], as well as for patients with cervical lymph node recurrence of thyroid cancer.

MWA functions by transmitting electromagnetic waves via a specialized microwave antenna. Its advantages over other MIT techniques include active ablation, shorter treatment duration, a larger ablation zone, reduced heat sink effect, decreased sensitivity to cooling effects from blood perfusion, the ability to uniformly ablate tumor targets adjacent to blood vessels, and compatibility with patients who have implants, such as pacemakers [5].

Despite the generally favorable prognosis of papillary thyroid carcinoma (PTC) and its responsiveness to conventional and MIT treatment, ~35% of patients may present with gross lymph node metastases [6]. Managing these metastatic lymph nodes remains controversial due to a lack of clear prognostic indicators. Available options include a new surgery with cervical nodes dissection, or radioactive iodine ablation. Recent studies have demonstrated promising outcomes using MIT such as MWA, thus gaining acceptance as a viable treatment modality for PTMC [7] and cervical metastases [8].

This study aims to present the inaugural experience in Ecuador of patients with benign and malignant TN, as well as cervical lymph node metastases, who underwent microwave thyroid ablation.

## Methods

### Study oversight

This case series study was approved by the Ethics Committee of the Universidad San Francisco de Quito, and all patients signed an informed consent form before undergoing MWA.

### Patients

From September 2022 to April 2023, 14 patients were included. The inclusion criteria for BNT were: (i) the longest diameter of the lesion is ≥2 cm; (ii) compressive or structural symptoms; (iii) cosmetic problems; (iv) only a single nodule of the thyroid gland; (v) refusal to undergo surgical treatment or intolerance for surgery; and (vi) normal thyroid functionality; (vii) two separate fine-needle aspirations (FNA) cytology biopsies.

Whereas patients with PTMC needed to fulfill the following criteria: (i) no lymph node metastasis (LNM) or extrathyroidal extension (ETE) on US pre-ablation; (ii) ineligibility or refusal to undergo surgery. Finally, for patients with previous PTC and new cervical lymph node metastasis (LNM), the inclusion criteria were: (i) total thyroidectomy, (ii) a single LNM confirmed with FNA, (iii) patients who had contraindications for surgery or refused surgery, or refused to undergo further surgical resection, (iv) absent radioiodine uptake at post-therapeutic 131I whole-body scan, (v) no distant metastasis in the follow-up and with (vi) data on serum thyroglobulin (sTg) levels available.

### Equipment

A microwave generator, SABERWAVE ECO-200G, that can produce 1-100 W of power at 2450 MHz either continuously or in a pulse and flexible internally cooled 16-gauge thyroid antenna (3 mm exposed tip and 10 cm shaft length). 2.45GHz Water Cooled Microwave System is CE and FDA-approved, manufactured by Nanjing ECO Medical Instrument Co, Ltd. (Nanjing, China).

### Pre-MWA procedure

The patients underwent new thyroid and cervical ultrasound to assess the volume, location, composition, echogenicity, margin, shape, echogenic foci, and blood flow. All images were collected by a single surgeon. The volume of the lesion was calculated using V = pabc∕6 (V: volume, a: maximum diameter, b and c: the other two perpendicular diameters). Also, a thyroid function test was performed before MWA and between 1 to 3 months post MWA.

Pain symptomatic score was evaluated with the visual analog scale (VAS) ranging from 0 (no pain) to 10 (severe pain). The cosmetic score was assessed by the surgeon and ranged from 1 to 4; 1 = no palpable mass, 2 = palpable but no visible mass, 3 = cosmetic problems on swallowing or neck extension, 4 = always visible.

The ultrasound provided information about the vascularization of the nodule. The type I vascularity refers to the complete absence of flow within the nodule; type II refers to perinodular flow; type III vascularity indicates intranodular flow with multiple vascular poles chaotically arranged, with or without significant perinodular vessels.

### MWA procedure

The patient was placed in supine position with the neck fully exposed, and local infiltration anesthesia with 2% lidocaine was administered. Hydrodissection was performed for lesions close to high-risk areas (near the trachea, esophagus, recurrent laryngeal nerve (LNR), carotid artery, and jugular vein). Then, an ultra-thin antenna #16G; shaft length 10 cm, and the MWA energy was initiated in 20–30 W. During the procedure, the moving shot technique was applied according to the volume, and location of the nodule. If a nodule was solid-cystic, the fluid portion was aspirated before MWA.

The patients were talking during the procedure to guarantee the integrity of LNR and helped to assess the patient’s pain. The ablation procedure is concluded when the echogenicity is changed completely. Post- MWA, the patients were monitored for 2 h, using ice local compression.

### Follow-up

All the patients had follow-ups at 1, 3, 6, and 12 months after MWA. During the appointments, the surgeon evaluated the symptomatic and cosmetic scores, and he performed an ultrasonography to assess the volume and the vascularization of the nodule. Then the volume reduction rate (VRR) of the MWA area was calculated using the following formula: VRR (%) = ([initial volume–final volume] × 100%)/initial volume.

Patients with cervical metastatic lymph nodes from PTC were followed up with measurement of diameters of LNs and inhibited serum thyroglobulin (s-Tg) at 1, 3, 6, and 12 months.

### Outcomes

For BTN and the PTMC, the primary outcome, volume reduction success, was defined as >50% at 3 months, >65% at 6 months, and > 90% at 12 months. The secondary outcomes were the thyroid functionality, symptomatic, and cosmetic score. Additionally, for PTMC, the ontological outcome was defined as noCLN or distant metastasis at the end of the follow-up. Finally, for LNM, we considered the ontological outcome as volume reduction, decrease s-Tg levels and no new recurrence (CLN or high Tg) during the follow-up.

### Statistical analysis

Statistical analysis was performed utilizing SPSS 20.0. Descriptive statistics, including the mean ± standard deviation (SD), were employed to characterize both the nodule size and patient age. Additionally, volume changes pre- and post-MWA were assessed via the paired samples t-test.

## Results

### Demographic and TN characteristics

This cohort study comprised 14 patients, 11 BTN, 1 PMTC, and two cervical lymph node metastases. The mean age of the patients was 48.92 (SD 11.20), and 86% of them were female. Most of them (73%) were euthyroid. Eleven nodules were mainly solid, and one was predominantly solid. In terms of their location, 42% were on the right, 50% on the left thyroid lobe, and 8% were in the isthmus. Regarding the vascularity, most patients were type III (50%) or type II (41.7%). All patients with BTN had Bethesda II and the patient with PTMC had Bethesda V (Table 1).

**Table 1:** Baseline characteristics.

**Variable**	
**Sex (n = 14)**	
Female	85.7% (n = 12)
Male	14.3% (n = 2)
**Age at diagnosis (n = 14)**	
Mean (standard deviation)	48.92 (11.20)
**Residence (n = 14)**	
Coast	7.1% (n = 1)
Highland	85.8% (n = 12)
Amazonia	7.1% (n = 1)
**Employment (n = 14)**	
Domestic chores	36.4% (n = 4)
Student	7.1% (n = 1)
Labor	56.5% (n = 9)
**Education level (n = 14)**	
High school	54.5% (n = 7)
University	45.5% (n = 7)
**Thyroid function (n = 14)**	
Euthyroid	72.7% (n = 9)
Hypothyroidism	27.3% (n = 5)
**Nodule composition (n = 12)**	
Solid	91.6% (n = 11)
Predominantly solid	8.4% (n = 1)
**Vascularization (n = 12)**	
Type I	8.3% (n = 1)
Type II	41.7% (n = 5)
Type III	50.0% (n = 6)
**Laterality (n = 12)**	
Right lobe	41.7% (n = 5)
Left lobe	50.0% (n = 6)
Isthmus	8.3% (n = 1)
**Localization of the main nodule (n = 12)**	
Middle third	41.7% (n = 5)
Lower third	8.3% (n = 1)
Isthmus	8.3% (n = 1)
Almost the entire lobe	33.3% (n = 4)
Almost the entire lobe and isthmus	8.3% (n = 1)
**Methods of detection (n = 12)**	
Symptomatic nodule	54.5% (n = 7)
Incidental, asymptomatic patient detected in the image	45.5% (n = 5)
**TIRADS (n = 12)**	
TIRADS 3	50.0% (n = 6)
TIRADS 4	41.7% (n = 5)
TIRADS 5	8.3% (n = 1)
**Bethesda (n = 12)**	
Bethesda 2	91.7% (n = 11)
Bethesda 5	8.3% (n = 1)

Continuous variables are expressed as mean (standard deviation). Categorical variables are presented as proportions (%). Abbreviation: TI-RADS: Thyroid Imaging Reporting and Data Systems

### Treatment variables

The mean operation time was 7′ 45″ (4.04) with the use of local anesthesia (33%) and conscious sedation (67%). The output power of MWA was 28.33 (6.9) W. The hydro dissection performed in the patients was lateral (42%), medial (17%), and mixed (33%). During the ablation procedure, two patients experienced soft pain, and it was alleviated post-MWA. There was no dysphonia, bleeding, infection, skin blunts, or esophageal or tracheal lesions. All the patients underwent a single session of MWA (Table 2).

**Table 2:** Treatment parameters of microwave ablation (MWA).

**Variable**	
**Anesthesia (n = 14)**	
Local	33.3% (n = 6)
Local + sedation	66.7% (n = 8)
**Microwave machine (n = 14)**	
Energy (W)	28.33 (6.9)
Time	7′ 45″ (4.04)
**Hydrodiseccion (n = 14)**	
Yes, lateral (carotid hydrodissection)	41.7% (n = 5)
Yes, medial (hydrodissection of trachea)	16.7% (n = 2)
Yes, mixed (hydrodissection of carotid and trachea)	33.3% (n = 4)
None	8.3% (n = 1)
**Number of sessions**	
Single	8
Additional	0
**Complication intra-operative (n = 14)**	
Soft pain	16.7% (n = 2)
None	83.3% (n = 10)

Continuous variables are expressed as mean (standard deviation). Categorical variables are presented as proportions (%). Abbreviation: RFA: radiofrequency ablation; J: joules; W: watts

### Treatment efficiency

#### Benign thyroid nodule

**Figure 1:**
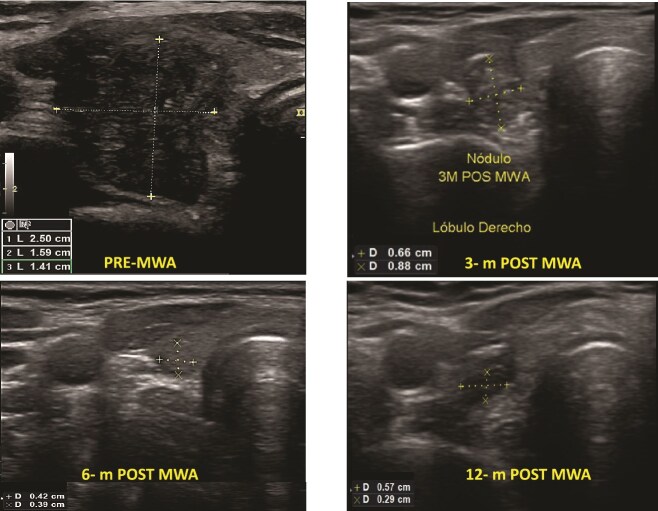
Benign thyroid nodules pre- and post-MWA (3, 6, and 12 months), see the changes in volume and the ultrasound aspect of the nodule.

Before MWA, the overall median volume was 1.54 (0.20–34.08) ml. After ablation, the 1-month, 3-month, 6-month, and 12-month median volumes were 1.80 (0.13–8.72) ml; 0.65 (0.01–12.23) ml, 0.11 (0.01–0.50) ml, and 0.05 (0.00–2.00) ml, respectively. The overall nodular volume reduced significantly after MWA treatment over time (*P* < .001). The VRR was 49%, 82%, 96%, and 97% at 1, 3, 6, and 12-month follow-ups, respectively (Table 3). Figure 1 shows an example of the change in volume of each patient’s nodule volume over time. The mean TSH in the 1-month follow-up was 2.84 (SD 1.61).

**Table 3:** Volume reduction ratio and TSH of BTN.

**Variables**	**Baseline**	**1 m**	**3 m**	**6 m**	**12 m**
Longest tumor diameter (mm)	17.0 (9–52) n = 11	20 (7–38) n = 9 *P* = .005	13.0 (6–37) n = 8 *P* = .04	6.0 (3–14) n = 8 *P* = .07	6.5 (2–18) n = 10 *P* = .015
Tumor volume (ml)	1.54 (0.20–34.08) n = 11	1.80 (0.13–8.72) n = 9 *P* = .003	0.65 (0.01–12.23) n = 8 *P* = .013	0.11 (0.01–0.50) n = 8 *P* = .03	0.05 (0.0–2.0) n = 10 *P* = .008
Volume reduction ratio (%)	-	−49.28 (−74; 64) n = 9	−81.75 (− 98; −64) n = 8	−96.0 (−99; −81) n = 8	−97.71 (−100; −90) n = 10
Grade of vascularization	8.3% (n = 8) 41.7% (n = 5) 50.0% (n = 6)	83.4% (n = 10) 8.3% (n = 1) 8.3% (n = 1)	91.7% (n = 11) 8.3% (n = 1) 50.0% (n = 0)	100% (n = 12) 0% (n = 0) 0% (n = 0)	100% (n = 12) 0% (n = 0) 0% (n = 0)
TSH	2.98 (1.61) n = 10	2.84 (1.1) n = 8			
Cosmetic score	2.5 (1.3) n = 11	2 (1.05) n = 9	1.4 (0.5) n = 8	1 n = 8	1 n = 11
Symptomatic score	5.8 (2.7) n = 11	4.1 (2.1) n = 9	1.9 (1.4) n = 8	0.3 (0.5) n = 8	0 n = 11

Data are Median [Interquartile Range]

#### Papillary thyroid microcarcinoma

**Figure 2:**
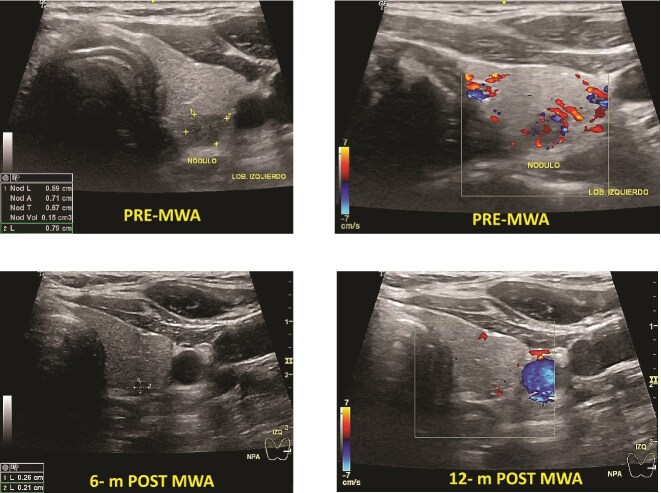
Papillary thyroid microcarcinoma pre- and post-MWA (6 and 12 months). Note that the nodule’s vanishing, making it hard to identify it by ultrasound.

One patient had PTC. The mean baseline volume was 0.13 mm. After ablation, the mean volume at 1-month follow-up was 0.05 ml, and after 12 months, it disappeared. There was no local tumor progression or lymph node metastasis (Fig. 2).

#### Cervical lymph node metastasis

**Figure 3:**
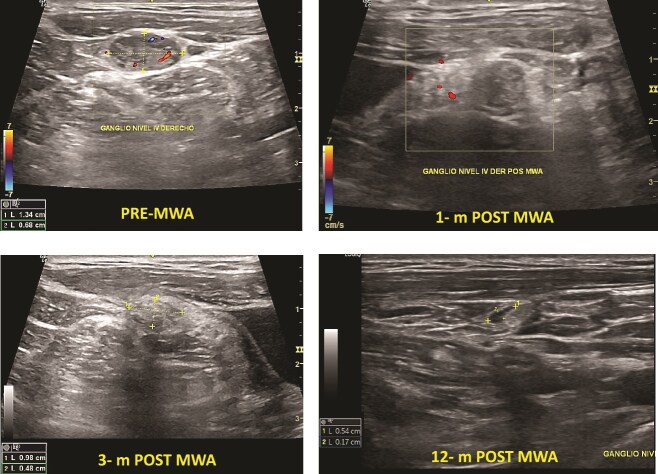
Cervical lymph node PTC metastasis pre- and post-MWA (1, 3, and 12 months).

Two patients had cervical metastasis of PTC confirmed by PAAF. Both were on the right side located on level 4. The longest diameter of the ablated LNs reduced from 13.0 (SD 4.0) mm to 5.0 (SD 3.1) mm (*P* < .001) at 6-month follow-up. Furthermore, the overall mean volume pre-MWA was 0.32 (0.2) ml. After ablation, the mean TN volume at 1-month, 3-month, 6-month, and 12-month were 0.22 (0.20) ml, 0.09 (0.10) ml, 0.45 (0.05), and 0.03 (0,01) respectively. The average s-Tg level pre-MWA was 10.5 (SD 7.0) ng/ml with anti-Tg <20, and it decreased to 1.23 (SD 1.1) ng/ml at 6-month and 1.12 ng/ml at 12-month follow-up (Fig. 3). There was no progression of CLN, distant metastasis, or high Tg during the follow-up.

#### Symptom score and cosmetics score

The symptom score and cosmetics score were significantly reduced at 6 months and were stable at 1-year post-operation (*P* < .05).

## Discussion

Microwave ablation represents an innovative and widely embraced minimally invasive therapeutic approach on a global scale. In this study, 14 patients were treated with MWA. The one-year follow-up results of the study showed that MWA may be effective and safe for the treatment of BTN, PMTC, or metastatic cervical lymph nodes in patients in Ecuador.

Microwave ablation was employed in the treatment of BTNs, yielding favorable therapeutic outcomes as evidenced in the follow-up [9]. The VRR in our study demonstrated substantial efficacy, reaching 98% at the 12-month follow-up. Like Du *et al* [10], and Liu *et al.* [11], the mean VR was 96.9% and 97% respectively in more than 3 years of follow-up. Additionally, Du *et al.* [10], in a retrospective long-term study of 148 BTN, reported that at 48 months follow-up the rate of complete disappear was 30%. In our study after one-year follow-up, seven (73%) BTN were completed absorbed and completed disappeared.

While the role of MWA in malignant thyroid lesions is still evolving, emerging evidence supports its use in select cases [ 12]. Cao *et al.* [13], in a retrospective study of 34 patients with isthmic PTC treated with MWA, reported tumor disappearance in 70.6% of cases. Similarly, Wu *et al.* [14]. demonstrated in a retrospective study of 106 patients with PTC close to the thyroid capsule that MWA was successful in all cases, with 70.0% of nodules disappearing and no local recurrences. Notably, voice changes were the only reported complication (5.7%), all of which resolved within six months. Furthermore, Han *et al.* [15] found that MWA is a safe and effective treatment for low-risk PTMC, with low local tumor progression rates and minimal complications. We presented one patient with unifocal PTMC, who had a VR of 98% after 1-year follow-up, without local tumor progression or cervical lymph node metastasis.

Regarding the cervical node’s metastases, we performed MWA in two patients who underwent thyroid surgery one year before MWA. They reduced in size and the mean serum thyroglobulin (Tg) level significantly decreased post-ablation). These findings are aligned with Cao *et al* [14]. study where they studied 14 patients with 38 cervical metastatic LNs and showed a significant reduction of the maximum diameter and Tg serum level.

In this study, two patients reported mild complication reportedly mild cervical pain while conducting MWA. We did not have major complications. However, Zhao *et al.* [15], in a retrospective study of 53 patients treated with MWA, reported hoarseness, a major complication, with an incidence of 1.89%. Also, Wu *et al* [16], reported that 2.7% (n = 2/75) of patients developed this complication. We believe that the main reasons of our low complication rate were the use of hydrodissection to prevent nerve injury during prolonged ablation and repeating this adjunctive as needed and the use of the doppler ultrasound.

Currently, we do not have patients with recurrence. Du *et al.* [10], reported a recurrence rate of 1.35%, however, Cheng *et al.* [17], in a prospective multicenter study of 649 BTN showed a higher recurrence rate of 8% in a longer follow-up. We probably did not find any recurrence as our follow-up is limited to 1 year.

This study has some limitations, including a small sample size and short follow-up data. However, it also has strengths, such as being the first experience of MWA in Ecuador, providing patients with a minimally invasive option to treat thyroid disease without removing the thyroid gland.

## Conclusion

MWA, as a minimally invasive technique, has demonstrated safety and efficacy in the treatment of BTN, low-risk PTMC, and lymph node metastases (LNM). MWA is associated with faster treatment times and the ability to create a larger, more uniform ablation zone, making it particularly advantageous for nodules located near blood vessels. Nonetheless, additional comprehensive studies with long-term follow-up are necessary in the Latin American population to further validate and expand the application of this therapy, particularly in cases of PTMC and LNM.
